# Home-based guidance training system with interactive visual feedback using kinect on stroke survivors with moderate to severe motor impairment

**DOI:** 10.1186/s12984-024-01479-7

**Published:** 2024-10-22

**Authors:** Hsuan-Yu Lu, Xiaoyi Wang, Chengpeng Hu, Cathy Choi-Yin Lau, Raymond Kai-Yu Tong

**Affiliations:** Department of Biomedical Engineering, The Chinese University of Hong Kong, Shatin, Hong Kong, SAR China

**Keywords:** Stroke, Home-based training, Joint angle, Center of mass, Visual feedback

## Abstract

The home-based training approach benefits stroke survivors by providing them with an increased amount of training time and greater feasibility in terms of their training schedule, particularly for those with severe motor impairment. Computer-guided training systems provide visual feedback with correct movement patterns during home-based training. This study aimed to investigate the improvement in motor performance among stroke survivors with moderate to severe motor impairment after 800 min of training using a home-based guidance training system with interactive visual feedback. Twelve patients with moderate to severe stroke underwent home-based training, totaling 800 min (20–40 min per session, with a frequency of 3 sessions per week). The home-based guidance training system uses Kinect to reconstruct the 3D human body skeletal model and provides real-time motor feedback during training. The training exercises consisted of six core exercises and eleven optional exercises, including joint exercises, balance control, and coordination. Pre-training and post-training assessments were conducted using the Fugl-Meyer Assessment-Upper Limb (FMA-UE), Fugl-Meyer Assessment-Lower Limb (FMA-LE), Functional Ambulation Categories (FAC), Berg Balance Scale (BBS), Barthel Index (BI), Modified Ashworth Scale (MAS), as well as kinematic data of joint angles and center of mass (COM). The results indicated that motor training led to the attainment of the upper limit of functional range of motion (FROM) in hip abduction, shoulder flexion, and shoulder abduction. However, there was no improvement in the active range of motion (AROM) in the upper extremity (U/E) and lower extremity (L/E) joints, reaching the level of the older healthy population. Significant improvements were observed in both left/right and superior/inferior displacements, as well as body sway in the mediolateral axis of the COM, after 800 min of training. In conclusion, the home-based guidance system using Kinect aids in improving joint kinematics performance at the level of FROM and balance control, accompanied by increased mediolateral body sway of the COM for stroke survivors with moderate to severe stroke. Additionally, spasticity was reduced in both the upper and lower extremities after 800 min of home-based training.

**Supplementary Information:**

The online version contains supplementary material available at 10.1186/s12984-024-01479-7.

## Introduction

Existing home-based training systems use games as the primary trend to enhance the level of motivation [1–5]. In addition, the training efficiency, in terms of repetitions, may be lower when utilizing the home-based training system without feedback [6]. If the fun environment is enriched and the correct movement is guided by visual feedback during training exercises, this approach would be a feasible training approach. Cloud computing technology has advanced recently, cloud-based networks can easily connect the training from home to the center, and therapists can adjust the training protocol remotely and access the training data. Two markerless systems for motion tracking 3D depth sensor technique and RGB camera system. 3D depth-sensing cameras, employing technologies such as stereo vision, time of flight, or structured light, are now capable of identifying 3D body segments. Notable examples include Kinect [7–11], ZED [12, 13], Intel RealSense [14]. Another type of system using markerless’s AI-driven motion capture technology with RGB cameras (*e.g*., Theia3D) [15] to construct the 3D skeletal models for tracking joint movements and balance control. In this work, we raise the following questions: Is it possible to have an interactive guidance system for home-based users during training, and what is the effectiveness of motor recovery after 800 min of home-based training using computer-guided visual feedback?

The home-based training approach [16–18] provides training for chronic stroke and benefits stroke survivors with an increased amount of training time and more feasibility in the training schedule, especially in patients with severe levels. The depth sensor-based training systems (*i.e.*, UINCARE Home + (UINCARE Corp., South Korea) [19, 20], MindMotion^®^ GO (Switzerland) [21], EvolvRehab (Spain) [22], LongGood TeleRehabilitation System (Taiwan) [23]) provide the visual feedback with specific symbols or targets to guide the user in achieving the tasks. However, these training approaches display virtual objects in a video game or demonstration videos without providing feedback on the user’s real-time motions. In this study, we developed a home-based guidance training system for stroke survivors, enabling to provide the RGB-depth sensor to capture 25 artificial anatomical landmarks to reconstruct the body skeleton and real-time visual feedback on users' body segment movements and joint angles during training.

The purpose of this study was to investigate the improvement in motor performance for stroke survivors with moderate to severe motor impairment after 800 min of training using the home-based guidance training system with interactive visual feedback. With the technology of depth sensors, home-based training with computer-guided motion guidance in real-time may help users improve motor performance with minimal therapist assistance, which can greatly enhance the flexibility to facilitate their training time schedule.

## Materials and methods

### Subjects

Twelve patients with chronic stroke (age: 63.8 ± 12.8 years [range from 41 to 83]; poststroke duration: 41.8 ± 33.4 months [range from 12 to 131]; training duration: 5.82 ± 2.92 weeks [range from 2.14 to 12.86]; affected side: 5 left/7 right; type: 8 ischemia/4 hemorrhage) were recruited from The Hong Kong Stroke Association. All subjects were matched to the included criteria of (1) being diagnosed with ischemic brain injury or intracerebral hemorrhage shown by magnetic resonance imaging or computed tomography after stroke onset, and (2) at least in the moderate motor impairment in upper-limb and lower-limb (FMA≦79) [24]. In addition, all subjects provided informed consent, which was approved by the Joint Chinese University of Hong Kong—New Territories East Cluster Clinical Research Ethics Committee before the experiment. The participants were excluded if they could not follow the instructions or independently conduct the rehabilitation training using the home-based guidance system.

### Home-based guidance system using kinect

**Fig. 1:**
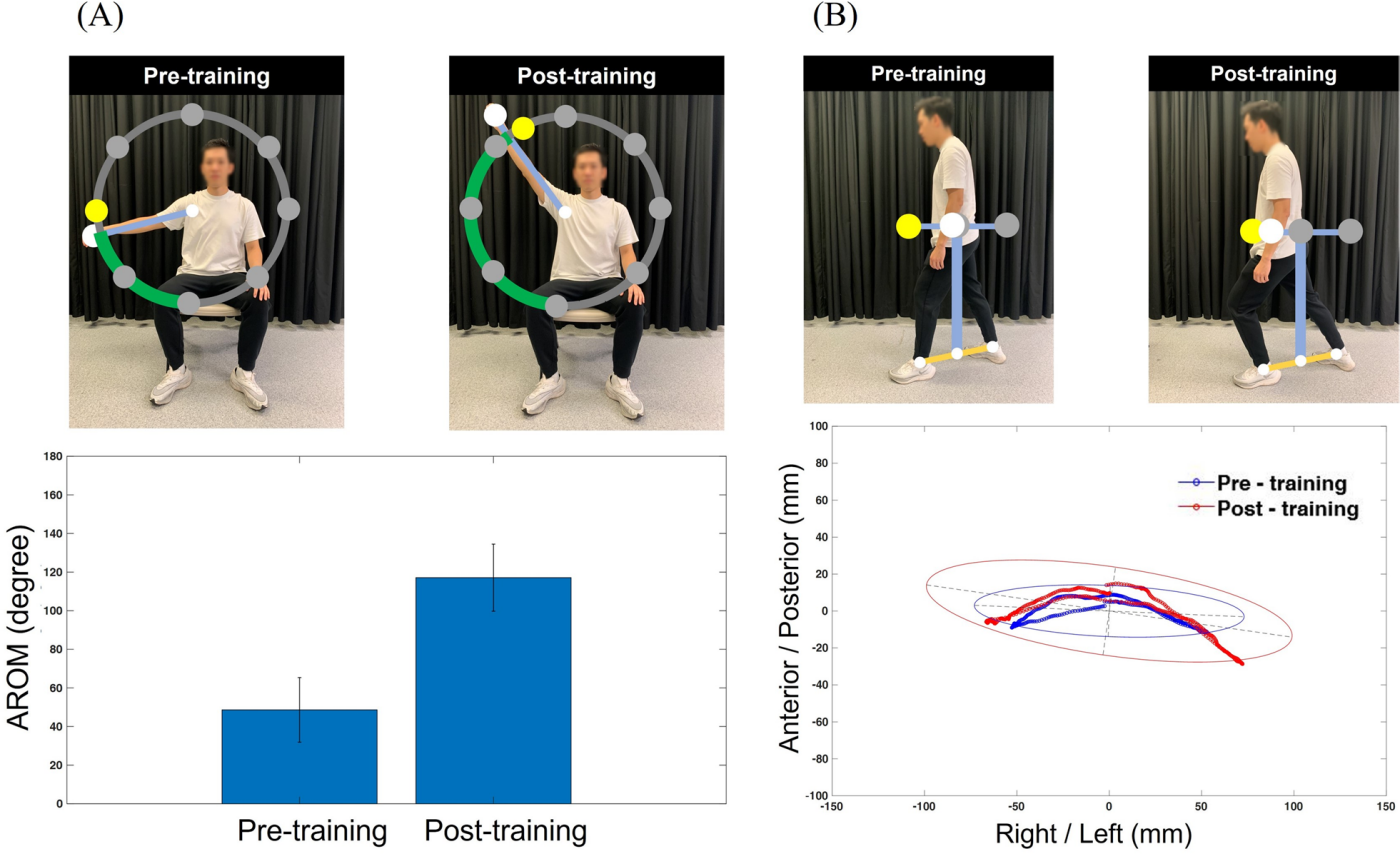
Illustration of real-time motor feedback by guiding interested joint/center of mass (white circle) to a specifically designed target (yellow circle) with corresponding kinematic performance in the training exercises of drawing a circle (Fig. 1a) and lunge (Fig. 1b) between pre- and post-training assessments using the home-based guidance system

A total of 25 artificial anatomical landmarks of the 3D skeletal model of the full human body, including body segments of the head, trunk, arm, forearm, hand, thigh, shank, and foot, were reconstructed using the body tracking software development kit (SDK) technology of Kinect for Windows SDK 2.0. This reconstruction was performed using a Microsoft Kinect V2 RGB-depth camera (sampling rate: 30 Hz, resolution: 512 pixels × 424 pixels, optimal distance of measurement: 0.5–4.5 m). The home-based guidance system was developed in C# programming with Microsoft Visual Studio (Visual Studio 2019, ©Microsoft Corporation, USA.). For each patient, the training exercise set consists of core exercises as well as personalized optional exercises. The core exercises of physical and occupational therapy included five exercises on shoulder flexion (*i.e.*, arm elevation on the sagittal plane), shoulder abduction (*i.e.*, arm elevation on the frontal plane), hip flexion (*i.e.*, thigh elevation on the sagittal plane), hip abduction (*i.e.*, thigh elevation on the frontal plane) and knee flexion (*i.e.*, shank elevation on the sagittal plane), and one balance control exercise of lateral weight shift. The therapist would select optional exercises for personalized training based on patient’s motor function recovery. The optional exercises included eleven exercises, such as drawing a circle, sit-to-stand, trunk rotation, etc. (details are shown in Table 1). This home-based system using a Kinect camera provided real-time motor feedback by guiding interested joints or the center of mass (COM) to a specifically designed target during the training exercises (Fig. 1a and b). The training exercises were designed on the 2D plane, but using the 3D coordinates reconstructed with the 3D human body skeletal model. Therefore, human body movement tracking would not be limited to joint motion parallel to the camera, and the correct movement patterns, assisted by motor guidance, could be provided. After finishing motor training, the personalized kinematic performance, animation, and score were provided in the daily report, and the total training time and average training time in the latest week were provided in the weekly report. All the reports were provided to both stroke survivors and the therapist. The therapist could update and manage the personalized optional exercises based on the weekly report once patients completed every three training sessions. The animation provides a video replay of the 3D human skeletal model at a frame rate of 30 fps during training exercises. Furthermore, it supports mouse control for 3D views, enabling zoom-in, zoom-out, and rotation.

**Table 1:** The training exercise set consists of core and personalized optional exercises, and the therapists updated the personalized optional exercises based on the weekly report

Core exercises (N = 6)	U/E joint exercise: (a) shoulder flexion, (b) shoulder abduction L/E joint exercise: (c) hip flexion, (d) hip abduction, (e) knee flexion Balance control: (f) lateral weight shift in sitting position
Optional exercises (N = 11)	U/E joint exercise: (a) clap hands over the shoulder, (b) elbow flexion, (c) drawing a square, (d) drawing a circle L/E joint exercise: (e) side step, (f) sit-to-stand Balance control: (g) trunk rotation in sitting position, (h) trunk rotation in standing position, (i) forward reach in sitting position, (j) lateral reach in sitting position Coordination: (k) contralateral U/E and L/E elevation

### Experimental procedure

Before motor training, the computer-guided visual feedback home-based system was installed on a mini-PC (OptiPlex 7080 Micro, Dell Technologies) at the patient’s home. The Kinect V2 camera was placed in front of the user at 1 m when using the home-based guidance system. In the beginning, experienced trainers helped patients become familiar with the procedures of the training exercises of the home-based system by following the demonstration video.

The amount of time spent [25, 26] influences the effects of addressing the impaired motor performance of a limited range of motion (ROM) and balance control in stroke patients. A minimum therapy input of 16 h (960 min) within the first 6 months was recommended to facilitate improvement in stroke survivors' activities of daily living (ADL) through augmented exercise [27], and four other studies demonstrated enhanced motor performance in upper limb range of motion (ROM) [28, 29], lower limb joints [30], and balance [31] through the use of home-based training systems. The duration of these interventions ranged from 600 to 720 min. Furthermore, both 600 min (10 sessions, 60 min each) and 1200 min (20 sessions, 60 min each) of training significantly improved balance recovery and resulted in faster muscle response after stroke training [32]. Hence, all patients underwent training sessions with a total of 800 min (3 sessions/week) based on the above literature to evaluate motor performance after training using our home-based guidance training system, and the duration of each training session ranges from 20 to 40 min which was determined by therapists.

### Clinical assessment for pre-training and post-training

All participants were arranged to test the clinical assessments for pre-training and post-training. The pre-training assessment was conducted before the first training within 2 weeks, and the post-training assessment was conducted within 1 week after finishing the last training session. In this study, the Fugl-Meyer Assessment (FMA), including Upper Limb (FMA-UE), Lower Limb (FMA-LE) and Total (FMA-Total), Functional Ambulation Categories (FAC), Berg Balance Scale (BBS), Barthel Index (BI) and Modified Ashworth Scale (MAS), was used to assess motor performance, motor skills, balance control, degree of independence and abnormal muscle tone in patients with poststroke hemiplegia. Therapists conducted and instructed all clinical assessments at the participant’s home.

### Quantified evaluation of kinematic performance

Besides clinical tests, pre- and post-training training assessments were also evaluated for kinematic performance. The tested motions for the affected side included the joint motion activities of (A) hip abduction, (B) hip flexion, (C) knee flexion, (D) arm elevation on the sagittal plane, (E) arm elevation on the frontal plane, and balance control of (F) lateral weight shifting from the neutral position as much as possible. The patient was asked to execute all the tested motions in a standing position. The pre- and post-training assessments were also arranged to evaluate the motor performance in joint motion and balance control activities. The calculated variables, including active range of motion (AROM), displacement of the COM, fitted ellipse area (EA) with COM, and axis length of the ellipse, are described in detail as follows.

#### Kinematics evaluation in joint motion

The AROM of joint rotation angles was used to evaluate the joint kinematics in the joint motion activities between pre-training and post-training assessments. The joint rotation angles were obtained from the 3D human-body skeletal model reconstructed via the official Microsoft SDK Beta2 version (Microsoft^®^ Visual Studio, Microsoft Corporation, USA.) [7]. In this study, the local coordinate system of the 3D skeletal model was determined with the positive x-axis directed to right, the positive y-axis directed superiorly, and the positive z-axis referred to posteriorly, described based on the spatial relation between the pose of the human body and the Kinect camera. The sagittal knee angle was calculated using in-plane angles between two adjacent vectors representing thigh/shank. The rotation angles in shoulder and hip joints were computed by using the Euler angle in z-x-y sequence, corresponding with flexion/extension, adduction/abduction, and internal/external, of the upper arm relative to the trunk and thigh relative to the pelvis, respectively. Compared to Vicon data, the accuracy of the mean value of root-mean-square error (RMSE) in joint angle measurement ranged from 3 to 17 degrees using the Kinect v2 camera [7].

#### Kinematics evaluation in balance control using COM

The body COM was calculated by weighting the 3D pose of 25 joints in the human-body skeletal model with Dempster’s coefficients, which provided an accurate body COM with a mean RMSE of 4.38 mm and mean Pearson correlation coefficient (CORR) of 0.94 comparable to that of the 3D infrared motion capture system [8]. This study used COM movements and human-body sway of the COM to quantify the balance control performance. The corresponding variables included right/left (R/L), superior/inferior (S/I), and posterior/anterior (P/A) displacements of the COM, major and minor axis lengths of the fitted ellipse with the COM, and fitted ellipse area (EA) with the COM. In addition, the fitted ellipse in the anatomical plane contains the COM trajectories with 95% confidence [33, 34].

### Statistical analysis

At first, Shapiro–Wilk test was used to test the normality in the baseline of FMA clinical assessments. Paired *t*-tests were used to test the differences in outcomes of FMA-UE, FMA-LE, FMA-Total, FAC, BBS, BI, and calculated variables of AROM, L/R, S/I and P/A displacements of COM, EA, and axis length of ellipse between pre- and post-training assessments. The nonparametric Wilcoxon signed-rank test was used to examine the differences in MAS between pre- and post-training assessments. The score of MAS 1^+^ ranges from MAS 0 to MAS 1, and MAS 1^+^ was substituted by a scale of 1.5 [35]. All comparisons were performed using SPSS 20.0 (SPSS, IBM, Armonk, New York, USA.). The significance level was set at α = 0.05. In addition, the power of clinical assessments for sample sizes of twelve patients between pre-training and post-training was evaluated by post hoc power analyses using GPOWER.

## Results

The *p*-values of normality tests in baseline motor performance of FMA-UE, FMA-LE, and FMA-Total were 0.513, 0.096, and 0.386, respectively. These results indicated that the data set of 12 stroke survivors showed no significant differences from the normal distribution. The post-training results, after 800 min of training at home, showed improvements in upper limb functions (FMA-U/E, AROM of shoulder joint), lower limb functions (FMA-L/E, AROM of hip and knee joints), as well as balance control performance (COM). In the clinical assessments, the mean (SD) of outcomes in FMA-U/E, FMA-L/E, FMA-Total, FAC, BBS and BI between pre- and post-assessment were 31.00 (12.77) and 43.58 (15.36), 18.08 (5.68) and 24.08 (5.6), 49.08 (17.2) and 67.67 (19.52), 4.92 (0.51) and 5.5 (0.8), 43.25 (10.56) and 46.0 (7.3), and 15.83 (2.95) and 17.17 (2.98), respectively (Table 2). The outcomes of clinical assessments showed significantly increased scores in post-assessment compared with pre-assessment, except for BBS (Table 2). Moreover, the effect size and power results in clinical assessments between pre-training and post-training are shown in Table 1. Most results showed a large effect size in the sample sizes of twelve with *α* levels of 0.05. Compared with the pre-assessment and post-assessment using the MAS, the results demonstrated significant decreases in spasticity scores for the upper extremities, lower extremities, and the total score (Table 3).

**Table 2:** Means (standard deviations) of the outcomes for patients with poststroke hemiplegia between pre- and post-training assessments after 800 min training in Fugl-Meyer Assessment of Upper Limb (FMA-UE), Fugl-Meyer Assessment of Lower Limb (FMA-LE), Fugl-Meyer Assessment in total (FMA-Total), Functional Ambulation Categories (FAC), Berg Balance Scale (BBS) and Barthel Index (BI)

	Pre-training	Post-training	*p* value	Effect size (Cohen's d)	Power	MDC/MCID distribution
FMA-U/E (max: 66)	31.00 (12.77)	43.58 (15.36)	< 0.001*	0.88	0.89	#[0, 0, 1 (8.3%), 11 (91.7%)] [36]
FMA-L/E (max: 34)	18.08 (5.68)	24.08 (5.6)	< 0.001*	1.06	0.96	#[0, 0, 6 (50%), 6 (50%)] [37, 38]
FMA-Total (max: 100)	49.08 (17.2)	67.67 (19.52)	< 0.001*	1.00	0.95	#[0, 0, 2 (16.6%), 10 (83.4%)] [36–38]
FAC (max: 6)	4.92 (0.51)	5.50 (0.8)	< 0.001*	0.83	0.85	[0, 4 (33.3%), 8 (66.7%)]*
BBS (max: 56)	43.25 (10.56)	46.00 (7.3)	0.137	0.29	0.25	[2 (16.6%), 1 (8.3%), 6 (50.2%), 3 (24.9%)] [37]
BI (max: 20)	15.83 (2.95)	17.17 (2.98)	0.002*	0.45	0.43	[0, 3 (24.9%), 9 (75.1%), 0] [39]

The asterisk indicated the significant difference with α = 0.05. The # symbol indicated the improvement reached the MDC/MCID level

MDC (minimal detectable changes), MCID (minimal clinically important difference). MDC/MCID distribution: number (percentage) of four levels [mild degeneration, no improvement, mild improvement, clinically significant improvement]. mild degeneration: -MDC/-MCID level < change < 0, no improvement: change = 0, mild improvement: 0 < change < MDC/MCID, clinical meaning improvement: change ≧ MDC/MCID

*No MDC/MCID in FAC, number (percentage) is listed in [mild degeneration, no improvement, improvement]

**Table 3:** The outcomes of the Modified Ashworth Scale (MAS) between pre- and post-training assessments, including flexor and extensor muscle tone for hip, knee, ankle, elbow, and wrist joints

	Pre-training	Post-training	*p* value
Hip Extensor	0.71 (1.18) [0, 0, 1, 0, 0, 0, 1, 1.5, 0, 4, 1, 0]	0.42 (1.16) [0, 0, 0, 0, 0, 0, 0, 1, 0, 4, 0, 0]	0.1250
Hip Flexor	0.50 (1.17) [0, 0, 1, 0, 0, 0, 0, 1, 0, 4, 0, 0]	0.42 (1.16) [0, 0, 0, 0, 0, 0, 0, 1, 0, 4, 0, 0]	1.0000
Knee Extensor	%1.%2 (1.11) [0, 1, 1, 0, 0, 0, 1, 1.5, 1, 4, 1.5, 1]	0.67 (0.89) [0, 1, 0, 0, 0, 0, 1, 1, 0, 3, 1, 1]	0.0625
Knee Flexor	0.58 (1.16) [0, 0, 1, 0, 0, 0, 0, 1, 0, 4, 0, 1]	0.33 (0.89) [0, 0, 0, 0, 0, 0, 0, 1, 0, 3, 0, 0]	0.250
Ankle Plantar-flexor	1.25 (1.27) [3, 0, 1., 5 0, 0, 1, 1, 1.5, 0, 4, 1, 2]	1.21 (1.27) [3, 0, 1.5, 0, 0, 1, 1, 1, 0, 4, 1, 2]	1.0000
Ankle Dorsi-flexor	1.25 (1.34) [3, 0, 2, 0, 0, 1.5, 1, 1.5, 0, 4, 0, 2]	1.17 (1.34) [3, 0, 2, 0, 0, 1, 1, 1, 0, 4, 0, 2]	0.5000
Elbow Flexor	0.71 (0.92) [1, 0, 0, 0, 0, 0, 1, 1, 1.5, 3, 1, 0]	0.50 (0.90) [0, 0, 0, 0, 0, 0, 1, 1, 0, 3, 1, 0]	0.5000
Elbow Extensor	0.79 (0.99) [0, 0, 1.5, 0, 0, 0, 1, 2, 0, 3, 1, 1]	0.42 (1.00) [0, 0, 0, 0, 0, 0, 0, 2, 0, 3, 0, 0]	0.1250
Wrist Flexor	1.00 (1.65) [3, 4, 0, 0, 0, 0, 0, 1, 0, 4, 0, 0]	0.67 (1.15) [1, 3, 0, 0, 0, 0, 0, 1, 0, 3, 0, 0]	0.2500
Wrist Extensor	1.17 (1.53) [3, 3, 2, 0, 0, 0, 0, 2, 0, 4, 0, 0]	0.88 (1.21) [1, 3, 2, 0, 0, 0, 0, 1.5, 0, 3, 0, 0]	0.2500
U/E component	3.67 (4.18) [7, 7, 3.5, 0, 0, 0, 2, 6, 1.5, 14, 2, 1]	2.46 (3.66) [2, 6, 2, 0, 0, 0, 1, 5.5, 0, 12, 1, 0]	0.0039*
L/E component	5.29 (6.53) [6, 1, 7.5, 0, 0, 2.5, 4, 8, 1, 24, 3.5, 6]	4.21 (6.02) [6, 1, 3.5, 0, 0, 2, 3, 6, 0, 22, 2, 5]	0.0078*
Total	8.96 (10.26) [13, 8, 11, 0, 0, 2.5, 6, 14, 2.5, 38, 5.5, 7]	6.67 (9.30) [8, 7, 5.5, 0, 0, 2, 4, 11.5, 0, 34, 3, 5]	0.0020*

The asterisk indicated the significant difference with *α* = 0.05

Values are mean (SD) [all data]. U/E component: elbow flexor and extensor, wrist flexor and extensor. L/E component: hip flexor/extensor, knee flexor/extensor, ankle dorsi-flexor/plantar-flexor

**Fig. 2:**
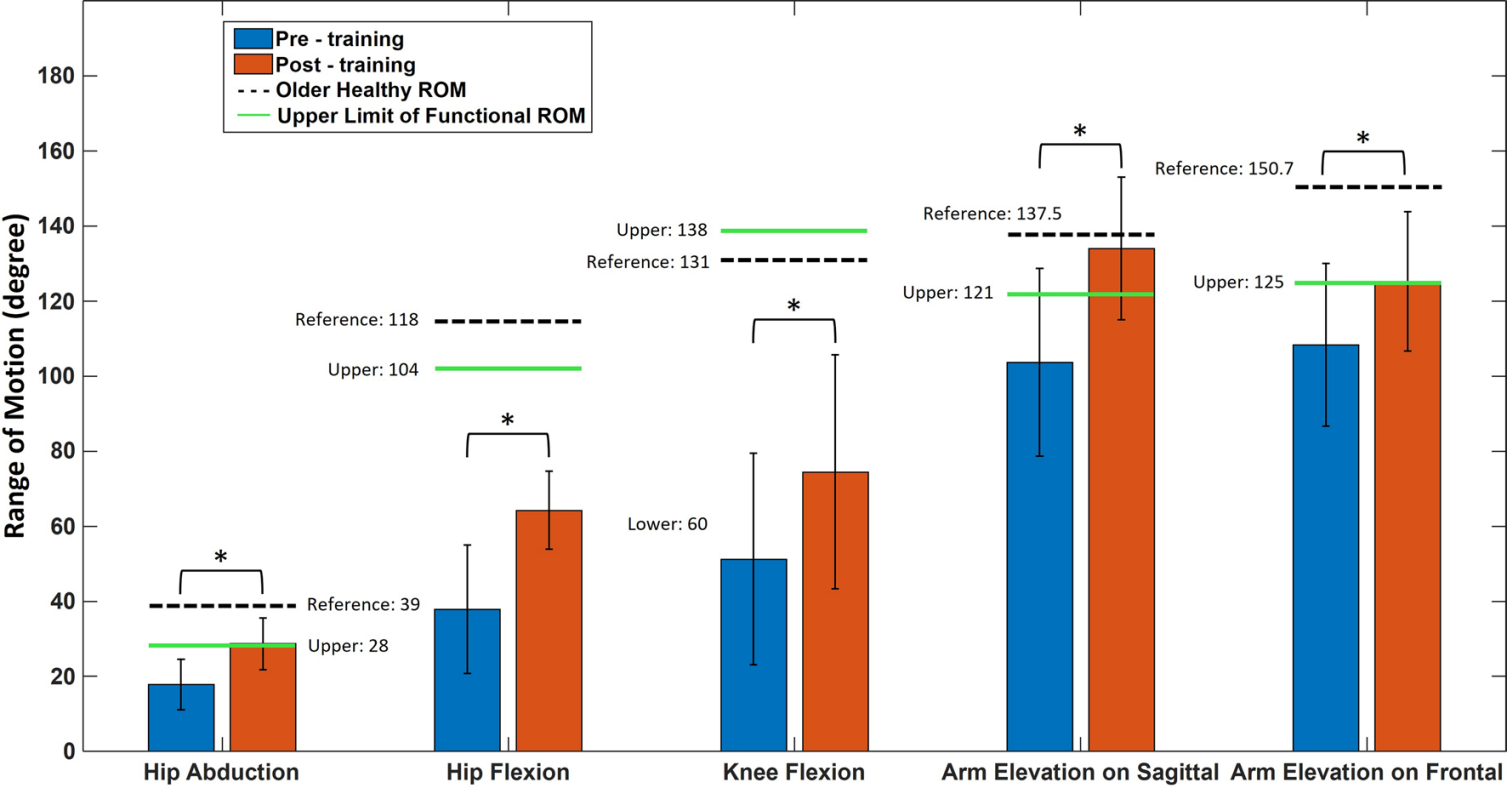
Mean and standard deviations between pre- (blue bar) and post-training assessments (red bar) of rotation angle in active range of motion (AROM) for hip abduction, hip flexion, knee flexion, shoulder flexion and shoulder abduction. The black dashed line indicated the AROM in the population of older healthy individuals [40, 41], and the green line showed the upper limit functional ROM for the performance of all activities of daily living (ADLs) [42–55]. The asterisk indicated the significant difference with *α* = 0.05

For the kinematic performance in joint motion, the mean (SD) AROM of the rotation angle in hip abduction, hip flexion, knee flexion, shoulder flexion and shoulder abduction between pre- and post-training assessments were 17.79 (6.72°) and 28.63 (6.9°), 37.83 (17.09°) and 64.23 (10.33°), 51.21 (28.2°) and 74.47 (31.14°), 103.68 (24.99°) and 133.98 (19.03°), 108.36 (21.65°) and 125.25 (18.58°), respectively (Fig. 2). The AROM of rotation angles for all joint motion exercises were significantly greater in post-training than in pre-training (Fig. 2). In addition, AROM in hip abduction, shoulder flexion and shoulder abduction after motor training achieved the upper limit of functional ROM (FROM), while no AROM in U/E and L/E joints achieved the level of the older healthy population (Fig. 2).

**Fig. 3:**
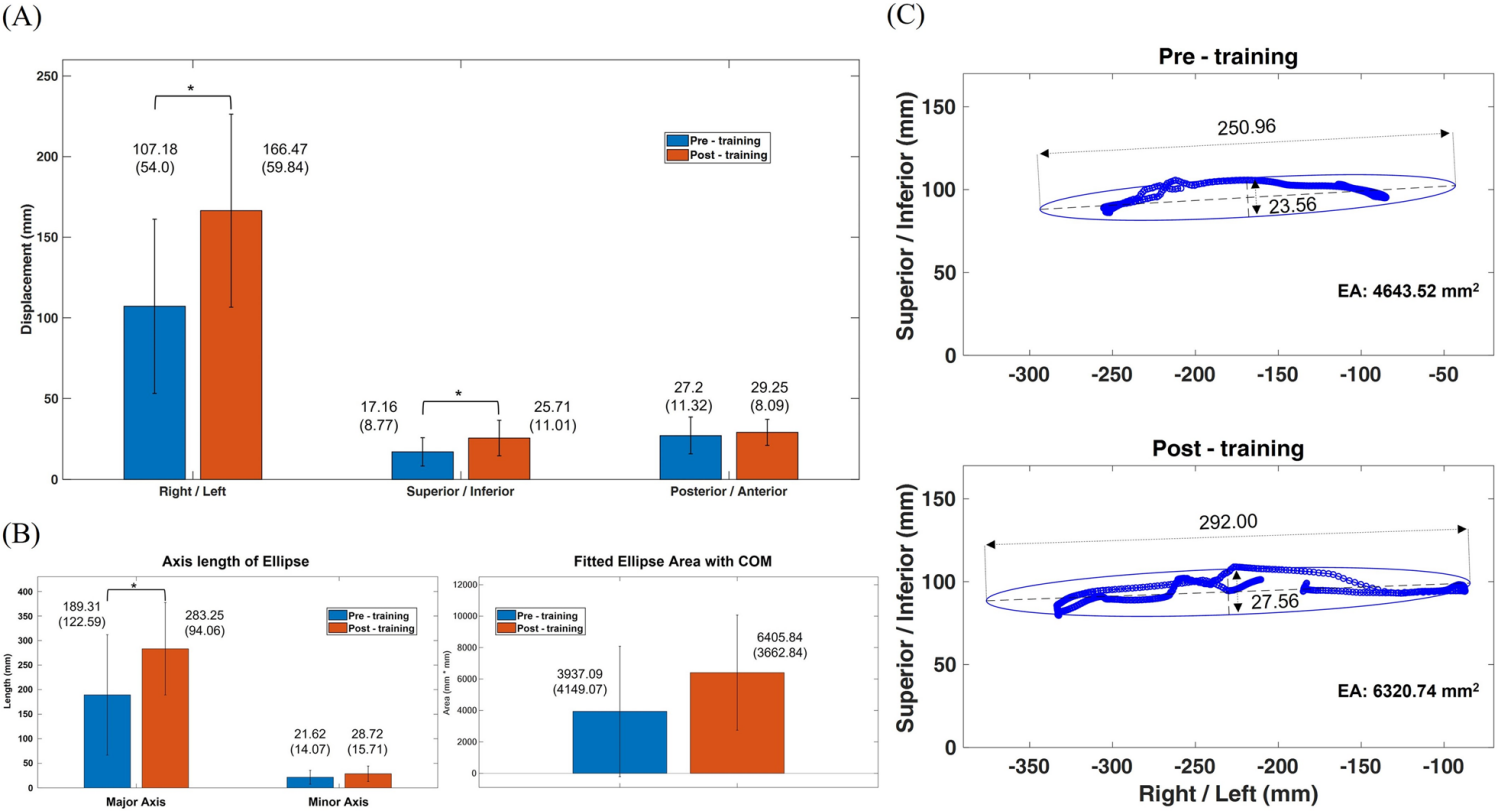
Mean and standard deviations between pre- (blue bar) and post-training assessments (red bar) of right/left (R/L), superior/inferior (S/I) and posterior/anterior (P/A) displacement of center of mass (COM) (Fig. 3a), and parameters of axis length and ellipse area (EA) obtained by fitting the trajectories of COM in R/L and S/I components with 95% confidence (Fig. 3b) during balance control task of lateral weight shifting. The asterisk indicated the significant difference with *α* = 0.05. In addition, an illustration of body sway with ellipses based on COM trajectories in R/L and S/I components for evaluating balance control performance. A typical subject showed the parameters of major (pre: 250.96 mm, post: 292.00 mm), minor axis length (pre: 23.56 mm, post: 27.56 mm) and EA based on COM with 95% confidence (pre: 4643.52 mm^2^, post: 6320.74 mm^2^) between pre- (blue) and post-training assessments (red) during 1 motion cycle of lateral weight shifting (Fig. 3c)

For the kinematic performance in balance control, the mean (SD) of R/L, S/I and P/A displacement of COM in lateral weight shifting between pre- and post-training assessments were 107.18 (54.0 mm) and 166.47 (59.84 mm), 17.16 (8.77 mm) and 25.71 (11.01 mm), 27.2 (11.32 mm) and 29.25 (8.09 mm), respectively (Fig. 3a). Both R/L and S/I displacements of COM were significantly greater in post-assessment than in pre-assessment, while no significant difference was found in the P/A component (Fig. 3a). The corresponding parameters of the major and minor axis lengths of the fitted ellipse with COM in the R/L and S/I components and fitted EA were 189.31 (122.59 mm) and 283.25 (94.06 mm), 21.62 (14.07 mm) and 28.72 (15.71 mm), and 3937.09 (4149.07 mm^2^) and 6405.84 (3662.84 mm^2^), respectively (Fig. 3b). The major axis length was significantly increased in post-training assessment compared with the pre-training assessment, while no significant differences were found in the EA or minor axis length (Fig. 3b). A typical subject showed the major (pre: 250.96 mm, post: 292.00 mm), minor axis length (pre: 23.56 mm, post: 27.56 mm) and EA (pre: 4643.52 mm^2^, post: 6320.74 mm^2^) between pre- and post-assessment during 1 motion cycle of balance control activity (Fig. 3c). In addition, the mean (SD) of the inclination angle (intersection angle between the major axis and the horizontal axis) of the fitted ellipse between pre- and post-assessment were 0.32 (5.48°) and 3.35 (4.57°), and no significant difference was found in this comparison.

## Discussion

Compared to pre-training, significantly increased AROM in shoulder, hip and knee joints were shown in post-training assessment after training using the home-based guidance system. In this study, the improvement in mean AROM between pre- and post-training for the shoulder joint exercises of arm elevation on the sagittal plane (i.e., shoulder flexion) and arm elevation on the frontal plane (i.e., shoulder abduction) were 30.3° and 16.89°. The corresponding AROM values in the L/E joint exercises of hip abduction, hip flexion, and knee flexion were 20.59°, 26.4°, and 23.26°, respectively (Fig. 2). For U/E kinematic performance, previous studies found a median AROM improvement of 20° in the shoulder joint for both shoulder abduction and shoulder flexion after receiving intervention with physical therapy (PT) and VR game training using a home-based training system [56]. These patients were arranged to receive the combination intervention of 20 sessions of PT and 20 sessions of Kinect-based VR game training, and the training frequency for PT and Kinect-based VR game training was 1 h a day with a duration of 4 weeks and 1200 min in total [56]. Another study demonstrated mean changes of 5.4° and 17.2° in AROM for shoulder abduction and shoulder flexion, respectively, after receiving 10 training sessions with 45 min/day duration and 450 min in total [57]. These studies utilized video game-based interventions, such as the KineLabs software platform [56] or exergames [57], as motor training protocols in home-based systems. For L/E kinematic performance, a previous study found that the improvement in median AROM for hip abduction, hip flexion, and knee flexion was 5°, 5° and 10°, respectively, after receiving game-based therapy with 10 sessions of training with 27–37 min/day duration (270–370 min in total) [58]. Compared to game-based therapy, it appeared that our therapist-prescribed guidance system for home-based training demonstrated the potential advantages of motor improvement in L/E and U/E joints. Moreover, the results of MAS indicated that spasticity showed a reduction in all subjects with one or more spastic muscles after training (Table 3).

These results are likely due to several factors related to the design of the therapist-prescribed guidance system for home-based training. First, the amount of training spent, including minutes provided, frequency and duration, are the vital factors influencing the effect on motor improvement for stroke survivors using the home-based guidance system [27]. This study utilized the amount of training spent of 800 min with 20–40 min/session and frequency of 3 sessions/week within 3.25 months. This choice was based on previous studies on stroke training, specifically the augmented-based systematic review [27] as well as research on virtual reality training using home-based systems [28–32]. Also, more intensive motor rehabilitation was recommended for stroke survivors to show better motor recovery within the subacute phase [59] (3–11 weeks after stroke) [60, 61]. Second, the interactive visual feedback with kinematics data (*i.e.*, joint center or center of mass) in real-time facilitates patients to perform exercise training correctly with improved motor performance with the assistance of motor guidance [62–64]. The type of training exercise differs between home-based therapy with therapist-prescribed exercises and game-based therapy. In clinical settings, patients trust the advice of professional therapists and follow the training protocols provided by therapists. In our home-based guidance system, we incorporate seventeen therapist-prescribed training exercises, including joint exercises, balance control, and coordination. Nevertheless, game-based therapy with an enriched and fun environment could also improve balance, motor performance and level of motivation in patients with stroke [65–67].

The analysis of COM movement has been commonly considered an essential variable to reveal the balance control mechanism underlying body stability during dynamic tasks in patients with motor dysfunction [68, 69], mobility recovery after stroke [70], people with deficits in movement [71] and the risk of falls in the elderly [72]. The human-body sway based on COM movements was also used to evaluate the balance control using Kinect as the clinical assessment [8, 73]. In addition, the fitted ellipse was used to describe the body sway based on COM trajectories in this study to qualify the displacement along the anatomical plane. The current results showed that significantly increased values were found in R/L and S/I displacements of COM as well as major axis length of the fitted ellipse with COM in R/L and S/I components during balance control activity of lateral weight shifting after motor training using the home-based guidance rehabilitation system. In this study, the improvements in mean displacements in R/L and S/I of COM between pre- and post-training assessments were 59.29 mm and 8.55 mm, respectively, and the corresponding parameter of major axis length was 93.94 mm (Fig. 3a and b). These findings indicated that therapist-prescribed guidance system for home-based training helped patients with stroke improve their balance control ability with increased COM displacements in the mediolateral and superoinferior directions and increased mediolateral body sway of the COM after motor training.

Compared to MDC (FMA-LE: 3.57 [37])/MCID (FMA-UE: 4.25 [36], FMA-LE: 6 [38]), the mean differences in FMA-UE, FMA-LE between pre-training and post-training were 12.58 and 6, which showing the greater or equal values. Moreover, mean differences in hip and knee AROM on the sagittal plane were 26.4° and 23.26°, showing greater improvement than minimal clinically important differences (MCID) of 5.81° [74] and 8.48° [75], respectively. By comparisons of the previous studies in the upper limit of the U/E and L/E rotation angles for the performance of ADL [42–55], the current results showed that patients with stroke achieved the upper limit of FROM level in hip abduction, shoulder flexion (*i.e.*, arm elevation on the sagittal plane) and shoulder abduction (*i.e.*, arm elevation on the frontal plane) after 800 min motor training by using the home-based guidance rehabilitation system with Kinect (Fig. 2). Nonetheless, the potential improvement in U/E and L/E joint kinematics between home-based training and the reference from the population in older healthy individuals [40, 41] still existed. Based on the baseline FMA-Total scores in pre-training assessment (Table 2), the stroke level of all the patients in this study was defined as ranging from moderate to severe (FMA-Total scores: 0–79) [24]. With the implicit outcomes of clinical assessments and kinematics evaluation, the home-based guidance system was proven to positively impact the improvement of motor performance in patients with moderate to severe stroke.

The current study aimed to investigate the improvement of kinematic performance for patients with stroke after motor training using the home-based training system with interactive motion guidance. The results showed that a home-based guidance rehabilitation system helps patients with moderate to severe stroke improve kinematic performance, reaching the level of MDC/MCID. Additionally, it improved FROM performance and increased body sway of the COM after training. These results may be further confirmed in the population of mild stroke patients after motor training using the home-based guidance rehabilitation system. The number of participants is relatively small. Further studies could recruit more subjects and be confirmed by randomized controlled trials (RCT) to provide more convincing clinical results. Table 2 showed that most patients showed mild improvement and clinically significant improvement in clinical assessments. More importantly, significant improvements were observed on the upper limb Fugl-Meyer Assessment (FMA) scores, with 91.7% showing clinically significant improvement and 83.4% showing clinically significant improvement on the FMA-Total. In addition, all patients exhibited an improvement (change > 0) in the active range of motion (AROM) of U/E and L/E joints. BBS did not exhibit significant differences in statistical analysis, with 75.1% showing improvement and 16.6% showing mild degeneration. Only 2 patients showed a level of mild degeneration in BBS, and less than 35% of patients exhibited no improvement in FAC, BBS and BI. It would be interesting to conduct further studies with a larger sample size, which could potentially enhance the training efficiency by incorporating personalized training protocols. In this study, home-based guidance training positively affected motor improvement after 800 min of training. To further investigate the effects of different training patterns, including home-based training, rehabilitation center-based training, and a hybrid combination of home-based and rehabilitation center-based training, developing stroke rehabilitation programs that incorporate guidance training systems at home and telemedicine would be beneficial.

## Conclusions

The guidance system for home-based users using Kinect provided interactive visual feedback and significantly improved clinical data on joint range of motion and balance control performance. The system had embedded six training exercises, and therapists could personalize the training program for each individual for 2 months of training with 800 min. The results showed that this Kinect-based home-based system helps patients with moderate to severe stroke improve joint kinematic performance at the level of FROM and balance control with increased mediolateral body sway of the COM. In addition, spasticity was reduced in both the upper and lower extremities after 800 min of home-based training.

## Supplementary Information

Additional file 1 (XLSX 30 KB).

## Data Availability

All data generated or analysed during this study are included in this published article [and its supplementary materials]. Information on this clinical trial can be found at https://clinicaltrials.gov/study/NCT04638218.
